# Ecological Dynamics of *Staphylococcus aureus* in Raw Ewe Milk Following Different Mastitis Treatment Protocols

**DOI:** 10.3390/antibiotics15040388

**Published:** 2026-04-10

**Authors:** Konstantina Fotou, Georgios Rozos, Konstantina Nikolaou, Vaia Gerokomou, Aikaterini Dadamogia, Sotiria Vouraki, Panagiotis Demertzis, Konstantoula Akrida-Demertzi, Natalia G. C. Vasileiou, Ioannis Skoufos, Athina Tzora, Chrysoula (Chrysa) Voidarou

**Affiliations:** 1 Laboratory of Animal Health, Food Hygiene and Quality, Department of Agriculture, School of Agriculture, University of Ioannina, 47100 Arta, Greece; grozos@uoi.gr (G.R.); knikolaou@uoi.gr (K.N.); v.gerokomou@uoi.gr (V.G.); kdadamogia@gmail.com (A.D.);; 2 Laboratory of Animal Science, Nutrition and Biotechnology, Department of Agriculture, School of Agriculture, University of Ioannina, 47100 Arta, Greece; svouraki@uoi.gr (S.V.); jskoufos@uoi.gr (I.S.); 3 Food Chemistry Laboratory, Section of Industrial and Food Chemistry, Department of Chemistry, University of Ioannina, 45110 Ioannina, Greece; pdemertz@uoi.gr (P.D.); kakrida@uoi.gr (K.A.-D.); 4 Faculty of Animal Science, University of Thessaly, 41110 Larissa, Greece

**Keywords:** *Staphylococcus aureus*, ovine mastitis, raw ewe milk, antimicrobial resistance (AMR), *mec*A, virulence-associated traits

## Abstract

**Background/Objectives:** *Staphylococcus aureus* (*S. aureus*) intramammary infection remains a major global dairy problem due to its contagious nature, its ability to persist and colonize teat/skin and mucosal niches, and the often-limited bacteriological cure achieved with antimicrobial therapy. Beyond udder health, it is relevant to public health because it can enter raw milk chains and serve as a reservoir for antimicrobial resistance determinants that may circulate between dairy animals and humans. **Methods:** We assessed *S. aureus*’ ecology in raw ewe milk from 75 sheep farms in Epirus (Greece) by sampling clinically healthy controls (group A) and clinical mastitis cases pre-treatment (group B), followed by resampling at the first post-withdrawal milking after penicillin/streptomycin treatment (group C1—therapeutic protocol 1), oxytetracycline treatment (group C2—therapeutic protocol 2), or enrofloxacin treatment (group C3—therapeutic protocol 3). **Results:** *S. aureus* detection was high and comparable across groups (A 23.0%, B 22.0–30.0%, C 20.0–22.0%), and paired analyses showed no significant pre–post shifts in detection/burden within therapeutic protocols (all *p* > 0.05). Nevertheless, persistence remained evident. The chromosomal gene *mec*A was detected in *S. aureus* strains in all groups, ranging from 13.6% in controls to 54.5% post-withdrawal in group C1, and was also present in the pre-treatment group. In paired sampling animals, *mec*A was mostly stable, with rare emergence or loss. Across antibiotic classes, within-animal resistance transitions were generally uncommon and non-significant (*p* > 0.05); β-lactam resistance was fully stable (*p* = 1.00). Descriptively, resistance to protein synthesis inhibitors tended to decline after therapy in protocol 1 and protocol 3, while protocol 3 showed post-treatment gains in fluoroquinolone resistance. By contrast, virulence-associated phenotype traits shifted after therapy: enterotoxigenicity increased post-withdrawal (especially in the C3 group), Staphylococcal Enterotoxin A (SEA) and Staphylococcal Enterotoxin B (SEB) appeared only post-therapy, Staphylococcal Enterotoxin D (SED) increased significantly in paired isolates (*p* = 0.002), and strong biofilm adherence increased (in C3, *p* = 1.5 × 10^−5^). **Conclusions:** The detection of *S. aureus* after therapy suggests that one possibility is that antimicrobial exposure may select for, or otherwise reshape, the residual intramammary population, rather than reliably eliminating it—an outcome that remains clinically relevant for udder health. Moreover, the persistence of *mec*A/methicillin-resistant *Staphylococcus aureus* (MRSA)-compatible profiles indicates that milk released to the food chain after withdrawal compliance may still harbor *S. aureus* with enhanced preservation capacity and significant food safety relevance.

## 1. Introduction

Ewe’s milk represents a small fraction of the global milk output (≈0.7%), yet it is disproportionately important from nutritional, economic, and cultural perspectives, particularly in regions where sheep husbandry and artisanal dairy processing are deeply embedded in local food systems [1,2]. Its high concentrations of protein, essential amino acids, minerals, vitamins, and bioactive lipids contribute to its nutritional density and help to sustain strong consumer demand, particularly for raw or minimally processed products [3,4]. Despite its value, both the milk yield and product quality can be markedly impaired by mastitis, a multifactorial disease in which *Staphylococcus* spp. are among the most frequently implicated etiological agents and are key determinants of clinical severity in small ruminants [5]. The genus *Staphylococcus* comprises more than 70 recognized species [2,6]. Staphylococci commonly colonize the skin and mucosal surfaces of humans and animals, including healthy sheep, but they can also cause infections, ranging from mild, localized disease to severe, life-threatening conditions [7,8]. Traditionally, they are classified as coagulase-positive (e.g., *Staphylococcus aureus*, *Staphylococcus hyicus*) or coagulase-negative (e.g., *Staphylococcus epidermidis*, *Staphylococcus haemolyticus*) [9]. The transition of staphylococci from commensal colonizers to mammary pathogens is shaped by interacting farm- and host-level determinants. These determinants include housing and hygiene, biosecurity, milking routines, climatic stressors, and breed-associated traits such as the teat morphology, susceptibility to intramammary infection, litter size, and lactation duration [10]. Among coagulase-positive staphylococci, *S. aureus* remains the most clinically consequential pathogen in dairy ruminants and a leading cause of both clinical and subclinical mastitis. In sheep, *S. aureus* is frequently associated with severe intramammary infection, with important consequences for ewe welfare, milk yields, and downstream lamb performance and survival. Beyond its role in udder health, *S. aureus* is also relevant to raw milk quality and safety, because isolates may express functional traits that support persistence in the mammary niche. In some cases, they carry genes encoding classical staphylococcal enterotoxins that are implicated in foodborne intoxication. From an ecological perspective, persistence and transmissibility are influenced not only by host and management factors but also by measurable strain-level phenotypes. For instance, hemolytic activity, extracellular enzymatic capacities, and biofilm-forming abilities can facilitate colonization, microenvironmental adaptation, and tolerance to antimicrobial exposure. Therefore, profiling these phenotypes provides an experimentally grounded way to describe the virulence potential of *S. aureus* populations in raw ewe milk, regardless of their clinical manifestation.

The treatment of ovine mastitis typically relies on intramammary therapy, often combined with systemic antimicrobials, ideally guided by pathogen identification and antimicrobial susceptibility testing. Despite the central role of small-ruminant farming in Greece, contributing approximately 15% of European small-ruminant milk production, the patterns of antibiotic use on farms, particularly in the treatment of mastitis, remain poorly characterized. The common clinical practice of “blind” antimicrobial treatment not only undermines therapeutic efficacy but also promotes the emergence and dissemination of antimicrobial resistance [11,12,13]. Accordingly, the surveillance of AMR in *S. aureus* from clinical mastitis and post-treatment milk is essential in evaluating therapeutic effectiveness, supporting evidence-based treatment choices, and tracking the emergence and spread of resistant lineages [7,14]. A central determinant of clinically important β-lactam resistance in *S. aureus* is methicillin resistance, mediated by the *mec*A gene. Consequently, *mec*A carriage is generally associated with elevated oxacillin/cefoxitin minimum inhibitory concentrations (MICs) and the reduced effectiveness of most β-lactams. However, the genotype–phenotype relationship is not invariably absolute, because expression can be heterogeneous and phenotypic categorization may be influenced by testing conditions and additional resistance mechanisms. For this reason, the interpretation of resistance ecology in *S. aureus* is strengthened by pairing phenotypic susceptibility profiles with *mec*A detection, particularly when assessing population shifts after antimicrobial exposure.

The aim of this study was to assess possible differences in the prevalence and dynamics of *S. aureus* in raw ewe’s milk collected under clinically defined conditions—healthy controls, clinical mastitis cases, and post-withdrawal sampling on the first milking—after three distinct mastitis treatment protocols. We further assessed the differences in the impact of the clinical status and of the therapeutic protocols on the antimicrobial susceptibility profiles of *S. aureus* isolates, including methicillin resistance, and evaluated functional traits relevant to virulence, persistence, and milk safety—namely hemolytic activity, extracellular enzymatic activity, biofilm-forming capacity, and the presence of the *mec*A gene among the groups.

## 2. Results

Samples (in total, 500) were classified into three principal groups according to the udder health status and sampling timepoint. Group A (100 samples) consisted of animals with clinically healthy udders and served as the control group. Group B (200 samples) consisted of animals with clinical mastitis, confirmed by compatible clinical findings and a California Mastitis Test (CMT) score > 2. Following field diagnosis, these animals were assigned by the attending veterinarians to one of three therapeutic subgroups according to the prescribed treatment regimen: group B1 (100 samples), penicillin G/dihydrostreptomycin; group B2 (50 samples), oxytetracycline; and group B3 (50 samples), enrofloxacin. After the completion of treatment and expiry of the corresponding withdrawal period, the same animals were resampled and reclassified as post-treatment groups C1, C2, and C3 (with the same numbers of samples as B1, B2, and B3, respectively), corresponding to B1, B2, and B3, respectively. This design generated paired pre- and post-treatment observations within the same animals for each therapeutic protocol. To ensure clarity and immediate understanding of the results, a concise summary table of the experimental groups, treatment allocation, and sampling timepoints is presented in this section (Table 1). While the full workflow of the study and the detailed grouping scheme are shown in Section 4 (Materials and Methods), this preliminary overview is added to support the direct, structured, and efficient interpretation of the results that follow.

Clarification: For brevity and consistency, throughout the manuscript, the following group designations are used: group A; group B, with subgroups B1, B2, and B3; and group C, with subgroups C1, C2, and C3.

Table 2 summarizes the distribution of *S. aureus* viable counts (log_10_ CFU/mL) across groups and timepoints. Culture results were interpreted as 0 = no growth, <1.00 = detected but below the quantification threshold (<10 CFU/mL), and ≥1.00 = quantified growth, allowing classification as no growth, low-level growth, and quantified growth. Overall, detection rates were similar between controls (A) and pre-treatment mastitis samples (B groups), and post-withdrawal detection showed only modest shifts across protocols. Importantly, paired inferential analyses detected no statistically significant differences in *S. aureus* detection or burden category from pre-treatment to post-withdrawal within any treatment protocol (McNemar exact and paired categorical analyses; all *p* > 0.05), and the detection rates did not differ significantly between protocols at baseline or post-withdrawal (all *p* > 0.05). Among quantified positives (≥1.00), changes in log_10_ CFU/mL were treated as supportive and likewise did not yield statistically significant differences (*p* > 0.05). Nevertheless, the descriptive pattern suggests comparatively more favorable outcomes for protocol 3 (highest cure proportion and lowest persistence among baseline-positive animals) and a reduction in detected/quantified positives after protocol 1, whereas protocol 2 showed little apparent change. These trends should be interpreted cautiously given the limited number of baseline-positive animals in Protocols 2 and 3 and the absence of statistically significant differences.

**Figure 1:**
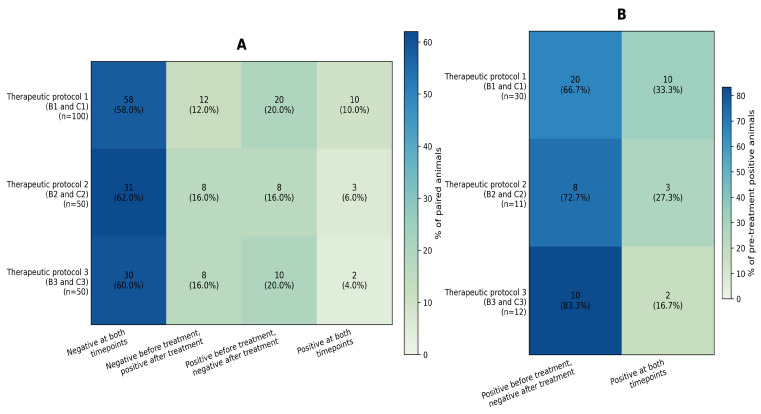
Paired *S. aureus* culture outcomes after treatment according to therapeutic protocol. Transition-state heatmaps summarize within-animal paired *S. aureus* culture outcomes across four categories: negative at both timepoints, negative before treatment but positive after treatment, positive before treatment but negative after treatment, and positive at both timepoints. (**A**) includes all paired animals within each therapeutic protocol, whereas (**B**) is restricted to animals that were positive before treatment.

The paired pre- and post-treatment culture outcomes for *S. aureus* are summarized in Figure 1A,B according to the therapeutic protocol. Animals were classified into four paired outcome categories between pre-treatment (group B) and post-withdrawal (group C) sampling: remained negative, negative before treatment but positive after treatment, positive before treatment but negative after treatment, and positive at both timepoints. Detection was defined as any recoverable *S. aureus* growth (<1.00 or ≥1.00 log10 CFU/mL), whereas negative denoted the absence of detectable growth. As shown in Figure 1A (all paired animals), for therapeutic protocol 1 (n = 100), 58 animals remained negative, 12 were negative before treatment but positive after treatment, 20 were positive before treatment but negative after treatment, and 10 remained positive. The corresponding values for therapeutic protocol 2 (n = 50) were 31, 8, 8, and 3 animals, respectively, whereas, for therapeutic protocol 3 (n = 50), they were 30, 8, 10, and 2 animals, respectively. Thus, post-withdrawal positivity was observed both in animals with persistent *S. aureus* isolation and in animals in which *S. aureus* was detected only at the post-withdrawal sampling point. As shown in Figure 1B (animals positive before treatment only), the transition from a positive to negative culture status was more frequent than persistent positivity across all therapeutic protocols. Nevertheless, persistent isolation remained evident in 10/30 animals (33.3%) under therapeutic protocol 1, 3/11 animals (27.3%) under therapeutic protocol 2, and 2/12 animals (16.7%) under therapeutic protocol 3. Overall, the figure highlights both the persistence of *S. aureus* after treatment and the occurrence of post-withdrawal detection among animals that were culture-negative at the pre-treatment sampling point. Thus, post-withdrawal positivity was observed both in animals with persistent *S. aureus* isolation and in animals in which S. aureus was detected only at the post-withdrawal sampling point; this latter finding may be attributable to new infections, reinfection, or the recovery of previously undetected low-abundance colonizing populations.

Table 3 presents the antimicrobial susceptibility profile (S/I/R; CLSI interpretive criteria) of all *S. aureus* isolates recovered from controls (group A; n = 22) and from clinical mastitis cases sampled pre-treatment (groups B1–B3) and at the first eligible post-withdrawal milking (groups C1–C3) under the three therapeutic protocols (group B1/C1: n = 30/22; B2/C2: n = 11/11; B3/C3: n = 12/7). Antibiotics are grouped by mechanism of action, enabling an assessment of both (i) agent-specific resistance frequencies and (ii) broader class-level resistance patterns across study groups.

Across the β-lactams, resistance was prominent but heterogeneous by agent and group. Penicillin G (BP) resistance was already substantial among controls, group A (8/22; 36.4%), and group B1 (10/30; 33.3%) but increased markedly in post-withdrawal group C1 (20/22; 90.9%) and was near-universal in groups B2 (11/11; 100%), C2 (10/11; 90.9%), B3 (12/12; 100%), and C3 (7/7; 100%). Ampicillin (AM) showed a broadly similar pattern (group A: 8/22, 36.4%; B1: 15/30, 50.0%; C1: 17/22, 77.3%), although AM resistance was not detected in C3 (0/7; 0%). Oxacillin (OX) resistance ranged from 14.3% to 40.9% in most groups (e.g., A: 5/22, 22.7%; C1: 9/22, 40.9%) but was absent in group B2 and group C2 (0/11 each), indicating protocol-associated heterogeneity in the phenotypic expression of methicillin resistance within this dataset. Cefalotin (CE) resistance was high in mastitis-associated groups (B1: 27/30, 90.0%; C1: 18/22, 81.8%), with substantial intermediate fractions in controls (I: 6/22, 27.3%) and C1 (I: 3/22, 13.6%), consistent with borderline susceptibility distributions for this agent.

Among the non-β-lactam classes, resistance was generally less common, although notable exceptions were identified. Vancomycin non-susceptibility (intermediate/resistant) was observed in several mastitis-related groups, including resistant isolates in group B1 (2/30; 6.7%), group C1 (2/22; 9.1%), and group C2 (2/11; 18.2%). By contrast, teicoplanin retained high activity across groups, with susceptibility being predominant and only occasional intermediate responses recorded. For protein synthesis inhibitors, erythromycin resistance was low to moderate (e.g., A: 6/22, 27.3%; B2: 3/11, 27.3%), clindamycin resistance remained low overall (group A: 2/22, 9.1%; B1: 1/30, 3.3%), and tetracycline resistance exhibited clear between-group variability (group A: 9/22, 40.9%; B1: 5/30, 16.7%; C2: 6/11, 54.5%; B3: 6/12, 50.0%). For fluoroquinolones, resistance was concentrated in protocol 3 post-withdrawal isolates: enrofloxacin (EN) resistance increased to 4/7 (57.1%) in group C3 and ciprofloxacin (CIP) resistance to 3/7 (42.9%) in group C3, while remaining undetected in most other mastitis groups (e.g., groups B1 and C1: 0% R for both EN and CIP). Trimethoprim/sulfamethoxazole resistance was present in control group A (3/22; 13.6%) and increased in group C3 (2/7; 28.6%). Fusidic acid resistance similarly increased in group C3 (2/7; 28.6%) compared with controls (2/22; 9.1%). Collectively, Table 3 indicates that resistance to several agents is widespread across the study population; however, the post-withdrawal isolate pool, particularly under protocol 3, includes higher proportions of resistance for specific drug classes. This supports the interpretation of therapeutic outcomes alongside downstream AMR hazard profiles at the point at which milk re-enters the food chain.

**Figure 2:**
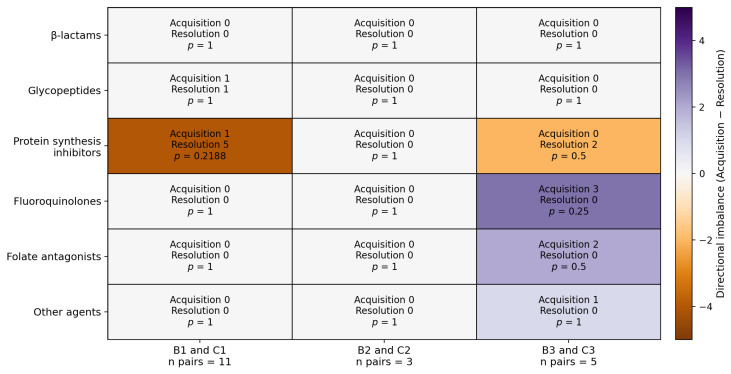
Paired pre- and post-treatment changes in class-level antimicrobial resistance within animals (B1–C1, B2–C2, and B3–C3), according to therapeutic protocol. For each class, isolates were categorized as resistant if they were resistant to at least one antibiotic within that class and as not resistant if all tested agents were susceptible or intermediate. Cell values indicate the number of transitions from not resistant to resistant (acquisition) and from resistant to not resistant (resolution), together with the exact McNemar test *p*-value. Cell color indicates the directional imbalance between acquisition and resolution.

Paired pre- and post-treatment comparisons between group B and group C were conducted within each therapeutic protocol to assess changes in antimicrobial resistance in the same animals before therapy (groups B1, B2, B3) and after the completion of therapy (groups C1, C2, C3) (Figure 2). For each antimicrobial class, the results were dichotomized using a class resistance index: an isolate was classified as resistant if it was resistant to at least one antibiotic within that class and as not resistant if all antibiotics in the class were categorized as susceptible or intermediate. Paired transitions were then summarized as “acquisition”, defined as a transition from not resistant before treatment to resistant after treatment, and “resolution”, defined as a transition from resistant before treatment to not resistant after treatment. In the heatmap, the cell color represents the balance between acquisition and resolution for each antimicrobial class and therapeutic protocol, with positive values indicating a greater number of acquisition events, negative values indicating a greater number of resolution events, and zero indicating no directional imbalance.

Overall, β-lactam resistance remained unchanged across all therapeutic protocols, as no transitions from not resistant to resistant (acquisition) or in the opposite direction (resolution) were observed between groups B1 and C1, groups B2 and C2, or groups B3 and C3 (all *p* = 1.00).

The overall antimicrobial resistance burden of the *S. aureus* isolates was assessed using the multiple antibiotic resistance (MAR) index and a weighted MAR index, both calculated on a per-isolate basis across the 20 antibiotics tested (Table 4). Isolates from control animals (group A, n = 22) exhibited a moderate resistance burden, with an MAR index mean ± SD of 0.157 ± 0.111 and a median of 0.150 (IQR, 0.100–0.150), whereas the weighted MAR index had a mean ± SD of 0.120 ± 0.098 and a median of 0.097 (IQR, 0.053–0.156). When pre-treatment mastitis isolates were pooled (group B, n = 51), the overall MAR distribution was similar, with a mean ± SD of 0.175 ± 0.099 and a median of 0.150 (IQR, 0.100–0.250). The weighted MAR index was likewise comparable to that of the controls, with a mean ± SD of 0.111 ± 0.080 and a median of 0.083 (IQR, 0.056–0.135).

Across the protocol-specific groups, the resistance burden was heterogeneous. In therapeutic protocol 1, the post-treatment group C1 (n = 22) showed higher MAR values than the corresponding pre-treatment group B1 (n = 30), with an MAR mean of 0.207 versus 0.150 and median values of 0.200 versus 0.150, respectively. The weighted MAR index followed the same pattern, increasing from a mean of 0.091 in group B1 to 0.124 in C1, with corresponding median values of 0.079 and 0.111. Therapeutic protocol 2 was characterized by comparatively elevated MAR and weighted MAR values in both B2 (n = 10) and C2 (n = 11) relative to most other groups, suggesting a generally higher resistance burden in isolates associated with this regimen. In therapeutic protocol 3, group C3 (n = 7) exhibited the highest resistance burden in the dataset, with an MAR mean ± SD of 0.271 ± 0.125 and a weighted MAR mean ± SD of 0.277 ± 0.164; meanwhile, the weighted MAR median was 0.222 (IQR, 0.167–0.373). The corresponding pre-treatment group B3 (n = 11) showed lower values, with an MAR mean of 0.200 and a weighted MAR mean of 0.114. Taken together, these descriptive findings indicate variability in the resistance burden across groups, with the highest weighted MAR values observed in group C3 and a general tendency toward increased resistance burdens in several post-treatment groups compared with their matched pre-treatment groups.

Table 5 presents class-specific comparisons of antimicrobial resistance between controls (group A), pooled pre-treatment mastitis isolates (group B pooled = B1 + B2 + B3), and post-treatment groups (C1–C3). Isolates were classified as class-resistant when resistant to ≥1 antibiotic within a class (R vs. not-R). Differences in class resistance prevalence were assessed using Fisher’s exact test with Holm correction within each class. In parallel, a continuous within-class burden measure (class MAR = number resistant within class/number tested within class) was compared across groups using the Kruskal–Wallis (KW) test. At baseline, β-lactam resistance was highly prevalent among mastitis isolates and was significantly higher in B pooled samples compared with controls (A: 17/22, 77.3% vs. B pooled: 50/51, 98.0%; *p*-Holm = 0.0333, KW *p* < 0.001), indicating the enrichment of β-lactam resistance in the mastitis-associated population before therapy. In contrast, fluoroquinolone resistance was more frequent in controls than in pooled pre-treatment mastitis isolates (A: 5/22, 22.7% vs. B pooled: 1/51, 2.0%; *p*-Holm = 0.0250, KW *p* < 0.001), suggesting that fluoroquinolone resistance was not a dominant baseline feature among the pre-treatment mastitis isolates in this dataset.

Comparisons between pooled pre-treatment isolates and post-treatment groups identified the strongest divergence in C3. Specifically, fluoroquinolone resistance increased markedly in C3 relative to B pooled samples (1/51, 2.0% vs. 5/7, 71.4%; *p*-Holm = 0.0001065, KW *p* < 0.001), and resistance to “other agents” was also higher in C3 (5/51, 9.8% vs. 4/7, 57.1%; *p*-Holm = 0.0329, KW *p* < 0.001). The corresponding KW *p*-values support these class-level differences at the level of the resistance burden (class MAR), consistent with non-uniform, class-specific shifts across groups. Collectively, Table 4 indicates (i) the baseline enrichment of β-lactam resistance in mastitis isolates and (ii) a pronounced post-treatment increase in fluoroquinolone and other agent resistance in C3 compared with pooled pre-treatment isolates.

**Figure 3:**
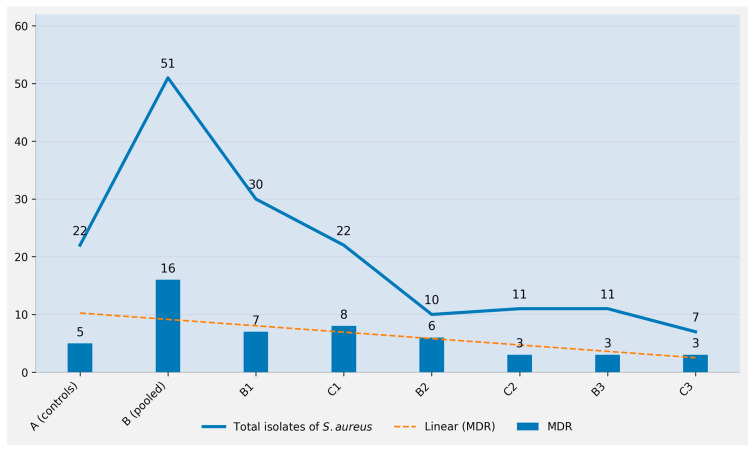
Prevalence of multi-resistance (MDR: ≥5 antibiotic resistances) among *S. aureus* isolates across experimental groups studied (control, pre-treatment mastitis, and post-treatment groups). Clarification: Bars show the absolute number of multi-resistant (MDR) isolates, defined here as isolates resistant to ≥5 of the 20 tested antibiotics, within each experimental group. The orange line indicates the total number of *S. aureus* isolates recovered per group (denominator), and numbers above bars/line represent the corresponding counts. The dashed line represents the linear trend in the number of MDR isolates across groups. Group B (pooled) combines all pre-treatment mastitis isolates (B1 + B2 + B3).

Figure 3 summarizes the distribution of multi-resistance (MDR; resistance to ≥5 of the 20 tested antibiotics) among the *S. aureus* isolates across the experimental groups studied, presented as absolute MDR counts together with the total number of isolates recovered per group. In total, 35 out of 113 *S. aureus* isolates were classified as multi-resistant (31.0%). Of these MDR isolates, 16/35 (45.7%) were recovered from the pooled pre-treatment mastitis group [B pooled (combined pre-treatment mastitis group) = B1 + B2 + B3], corresponding to 16 MDR isolates among 51 total isolates in group B pooled (31.4%). MDR isolates were also detected across the remaining experimental groups, including the controls (group A: 5/22, 22.7%) and the protocol-specific pre- and post-treatment groups (B1: 7/30; C1: 8/22; B2: 6/10; C2: 3/11; B3: 3/11; C3: 3/7), indicating that multi-resistant phenotypes were present throughout the study groups.

Table 6 presents the frequency of the main phenotypic methicillin resistance screening markers, oxacillin minimum inhibitory concentration (MIC) resistance, cefoxitin disk diffusion positivity, positivity in any oxacillin salt agar screening assay (OSAS positivity), and penicillin-binding protein 2a (PBP2a) detection, together with *mec*A genotype positivity and the study’s composite MRSA classifications (MRSA_screen and MRSA_confirmed) across the control group (group A) and the mastitis groups before treatment (groups B1–B3) and after treatment (groups C1–C3). The prevalence of individual markers varied markedly between groups, indicating heterogeneity in the distribution and phenotypic expression of methicillin resistance determinants. PBP2a positivity ranged from 18.2% in group A (4/22) to 72.7% in group C1 (16/22), while *mec*A positivity ranged from 13.6% in group A (3/22) to 54.5% in group C1 (12/22). Similarly, the phenotypic screening markers showed considerable variation, with oxacillin MIC resistance ranging from 0.0% to 40.9%, cefoxitin positivity from 16.7% to 33.3%, and OSAS positivity from 9.1% to 36.4%—findings that are consistent with the expected but incomplete concordance between genotypic and phenotypic methicillin resistance profiles in field isolates.

Using the composite classification, MRSA-confirmed isolates were detected in all experimental groups, ranging from 13.6% in controls (group A: 3/22) to higher proportions in mastitis-associated groups, including B1 (10/30; 33.3%), C1 (12/22; 54.5%), B2 (3/11; 27.3%), C2 (2/11; 18.2%), B3 (5/12; 41.7%), and C3 (2/7; 28.6%). The highest MRSA-confirmed proportion was observed in C1 (54.5%). Because these are group-level frequencies, differences between group B and group C should be interpreted as differences in the distribution of markers among isolates recovered at each sampling point, rather than as definitive evidence of the treatment-driven acquisition or loss of MRSA; such inference requires strain-level continuity.

Within the paired animal subset, the *mec*A status was predominantly stable between the pre-treatment and post-treatment sampling points. Persistent *mec*A positivity was recorded in 11 paired cases, newly detected *mec*A positivity in two cases, and loss of detectable *mec*A in three cases, while the remaining pairs remained negative at both timepoints. Where the *mec*A status changed, this was generally accompanied by concordant directional changes in one or more phenotypic methicillin resistance markers and/or in the composite MRSA classification. Nevertheless, a minority of paired profiles displayed genotype–phenotype discordance, consistent with variability in the expression and/or detectability of methicillin resistance markers in field isolates.

Table 7 summarizes the phenotypic enterotoxin profiles of *S. aureus* isolates as determined by the Staphylococcal Enterotoxin Reversed Passive Latex Agglutination (SET-RPLA) assay across the experimental groups. Overall, the enterotoxin pattern differed between the control, pre-treatment, and post-treatment groups. In the pre-treatment mastitis groups, Staphylococcal Enterotoxin A (SEA) and Staphylococcal Enterotoxin B (SEB) were absent, whereas Staphylococcal Enterotoxin C (SEC) and Staphylococcal Enterotoxin D (SED) predominated. After treatment, SEA and SEB became detectable in selected post-treatment groups, while overall enterotoxigenicity appeared more frequent, particularly in group C3. Across the mastitis-associated isolates, SEC and especially SED remained the dominant serotypes. Collectively, these findings suggest a shift in the enterotoxigenic phenotype after treatment, characterized mainly by broader toxin detectability and increased prominence of SED.

To further support the findings of the present study, detailed paired within-animal changes in phenotypic enterotoxin detection between the pre-treatment mastitis groups (B1–B3) and the corresponding post-treatment groups (C1–C3), stratified by therapeutic protocol and combined overall, are provided in Supplementary Table S1. For each toxin, paired outcomes were classified as persistent positivity, loss of positivity after treatment, gain of positivity after treatment, or persistent negativity. Changes were assessed using exact McNemar tests based on the discordant paired outcomes.

**Figure 4:**
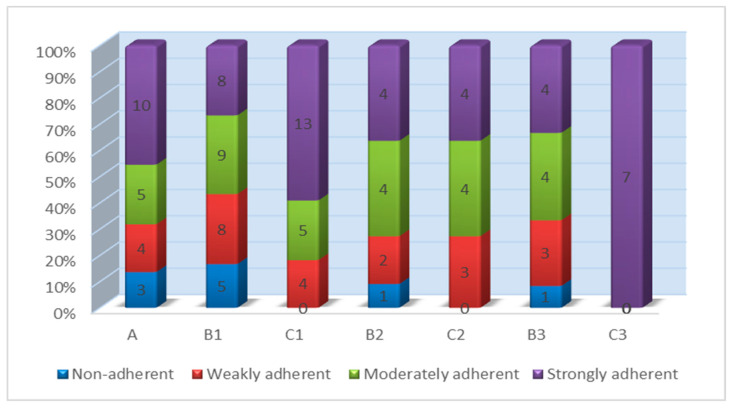
Distribution of biofilm adherence phenotypes by experimental group and paired pre–post change following therapy. Biofilm adherence was classified according to Christensen-type criteria using OD_570_ values relative to the optical density cut-off (ODc), as defined by the optical density of the reference negative control samples: non-adherent, OD_570_ ≤ ODc; weakly adherent, OD_570_ > ODc to 2 × ODc; moderately adherent, OD_570_ > 2 × ODc to 4 × ODc; strongly adherent, OD_570_ > 4 × ODc.

The distribution of biofilm adherence phenotypes across the study groups is shown in Figure 4, as determined by the microtiter plate adherence assay (OD_570_), interpreted relative to the reference negative control samples (ODNC). Overall, both the control group and the pre-treatment mastitis groups displayed heterogeneous adherence profiles spanning all biofilm categories. In contrast, the post-treatment groups showed a clear shift toward stronger adherence phenotypes, with reduced representation or the complete absence of non-adherent isolates. This pattern was most pronounced in C1 and especially in C3, indicating that post-treatment isolates were not depleted of biofilm-forming capacity but were instead enriched in strongly adherent phenotypes.

Paired within-animal comparisons were performed between the pre-treatment mastitis groups (B1–B3) and the corresponding post-treatment groups (C1–C3), using only animals with matching IDs. In total, 19 matched pairs were available (protocol 1: n = 11, protocol 2: n = 3, protocol 3: n= 5). Overall, the biofilm phenotype shifted toward stronger adherence after treatment: adherence increased in 17 of 19 animals, remained unchanged in two, and did not decrease in any case. In terms of OD-based classification, this corresponded to a shift from predominantly moderate adherence before treatment to predominantly strong adherence after treatment. This overall increase was supported by both the sign test (*p* = 1.5 × 10^−5^) and the Wilcoxon signed-rank test (*p* = 2.0 × 10^−4^). At the protocol level, protocol 1 showed the most consistent increase, with adherence rising in 10 of 11 matched animals and remaining unchanged in one, indicating a shift from mainly moderate to predominantly strong adherence after therapy. In protocol 2, all three matched animals showed increased adherence, consistent with a shift from weak to strong adherence, although formal statistical inference was limited by the very small sample size. In protocol 3, adherence increased in four of five matched animals and remained unchanged in one, again indicating a post-treatment shift toward strong adherence. Overall, the paired analysis indicates that post-treatment isolates were enriched in stronger biofilm-forming phenotypes rather than showing the attenuation of the adherence capacity.

## 3. Discussion

Mastitis in small ruminants is best understood as an inflammatory syndrome of the mammary gland, with substantial clinical and economic relevance, in which disease expression reflects a multifactorial, polyetiological interplay among host immunity, management, and environmental exposure [15,16,17,18,19]. Accordingly, infections may involve a single pathogen or mixed bacterial flora, and the isolation of one microorganism does not, by itself, establish exclusive causality. Within the raw milk continuum, the ecology of *S. aureus* in mammary secretions and raw milk is central to mastitis research and dairy safety, as the bacterium can simultaneously function as a contagious intramammary pathogen, persist within the teat–udder microbiological niche, and contribute to raw milk contamination with enterotoxigenic and/or antimicrobial-resistant strains [2,7,10,12,20,21]. In the infected mammary gland, *S. aureus* commonly exhibits persistence phenotypes, most notably biofilm-associated growth, which helps to explain recurrent/chronic infections and the frequent failure to achieve a bacteriological cure despite apparently favorable in vitro susceptibility [22]. From a sampling and interpretation standpoint, aseptic milk collection is essential because environmental sources (teat/udder skin, barn environment) and the sampler’s hands can introduce exogenous organisms and confound culture-based inference. However, even when contamination is rigorously minimized, isolation from mammary secretion can still reflect true biological ingress through the teat canal, since microorganisms on the teat surface can penetrate during or after milking, and the teat canal may remain functionally open for 1–2 h post-milking (or longer when the teat end is damaged), facilitating entry [23,24]. Collectively, these ecological realities—persistence, intermittent shedding, and a physiologically plausible entry route—mean that post-treatment milk can remain clinically and epidemiologically relevant for both udder health outcomes and raw milk hazard profiles.

This study investigated the ecology of *S. aureus* in raw ewe milk across clinically defined states—healthy controls, clinical mastitis before treatment, and the first eligible post-withdrawal milking—using a paired within-animal design across three field-applied antimicrobial protocols (penicillin/streptomycin, oxytetracycline, and enrofloxacin). By integrating the culture-based bacterial burden, antimicrobial susceptibility profiles (including *mec*A/MRSA-compatible markers), and functional traits associated with persistence and hazard, such as biofilm adherence and enterotoxin production, the study presents the broader characterization of isolates recovered after treatment as compared to a simple presence/absence assessment. Because strain-level continuity was not established, the post-withdrawal patterns observed cannot be attributed solely to within-host selection and may also reflect the persistence of the same strain, the emergence of previously undetected subpopulations, intermittent shedding around the culture detection threshold, or strain turnover/reinfection. Within these limitations, the results define the phenotypic profile of S. aureus isolates recoverable at the time when milk becomes legally eligible to re-enter the food chain.

Staphylococcal mastitis is a major cause of economic loss in small-ruminant production, particularly in Mediterranean systems [25,26]. Across both multifactorial surveillance studies and pathogen-focused investigations, *S. aureus* is consistently identified as a major ovine mastitis pathogen, although the reported prevalence varies substantially with the case definition, sampling strategy, and diagnostic thresholds [2,10,13,16,27,28]. High recovery has been documented in both clinical and subclinical disease, supporting efficient within-flock transmission and persistence in mammary reservoirs [29,30,31]. This is in line with our finding of high detection frequencies in both the controls and pre-treatment mastitis groups, suggesting a substantial reservoir of subclinical carriers and intermittent shedders.

At the same time, lower detection rates in other settings show how strongly the apparent prevalence is shaped by the local epidemiology, management, and methodology [28,32,33]. Chambers et al. [28], for example, linked low recovery in dairy sheep clinical mastitis to diagnostic and culture threshold effects, highlighting how single-timepoint cultures may underestimate infection dynamics under intermittent shedding. Beyond clinical sampling, studies of raw and bulk-tank milk further connect intramammary infection with contamination of the milk chain [2,34]. In particular, the high recovery reported in subclinical mastitis by Iancu et al. [31], together with detection in raw sheep milk and bulk-tank milk by Roșu et al. [2] and Giacinti et al. [34], supports the view that *S. aureus* may remain highly prevalent even when clinical mastitis is not the dominant visible phenotype. These observations are consistent with our findings and emphasize the dual significance of *S. aureus* as both a mastitis-associated pathogen and a potential milk chain hazard.

Overall, published studies indicate that statistically stable bacteriological outcomes are compatible with biological activity in endemic *S. aureus* systems, where reservoir carriage, intermittent shedding, reinfection, and culture sensitivity may reduce the detectability of treatment effects [26,28,29]. In such settings, paired outcome patterns may be more informative than simple prevalence contrasts. This interpretation is supported by evidence of efficient within-flock transmission, mammary persistence, and the recognized difficulty of complete post-treatment elimination [15,29,35,36,37,38].

In the present study, paired within-animal transitions in class-level antimicrobial resistance were infrequent and non-significant across all pre-treatment versus post-treatment comparisons (group B versus group C), indicating short-term phenotypic stability after therapy. β-Lactam resistance remained unchanged across protocols. Limited directional patterns were observed for protein synthesis inhibitors under therapeutic protocol 1 and for fluoroquinolones under therapeutic protocol 3, but these findings should be interpreted cautiously because post-treatment isolates may reflect selective pressure, strain replacement, or the detection of previously underrepresented subpopulations, rather than the persistence of the same strain. Inducible clindamycin resistance showed no consistent post-treatment increase [39]. The MAR, weighted MAR, and MDR patterns also indicated heterogeneity rather than a uniform post-treatment shift, with the clearest increase in group C3 and a smaller increase in group C1. This interpretation is consistent with data from bovine mastitis showing no immediate increase in resistance after intramammary therapy [40] and with the marked regional variability reported across sheep dairy systems [41,42,43], including studies from Sardinia [26], reports from the United Kingdom [30], raw milk samples collected in Romania [31], mastitis cases investigated in Jordan [33], and isolates recovered in Iran [44]. The fluoroquinolone-associated pattern observed here is also consistent with previous reports describing both the therapeutic efficacy of enrofloxacin and post-treatment increases in MIC values or resistance determinants after exposure [45,46]. Overall, the present results indicate that short-term post-treatment resistance profiles in ovine *S. aureus* may remain stable at the class level, while selective changes may still occur in the composition of the recoverable isolate population. Similarly, UK surveillance reported generally low AMR in *S. aureus* isolates from ovine mastitis, while noting that larger, adequately powered studies are needed to guide control strategies and to detect emerging resistance trends reliably [30].

Across small-ruminant dairy systems, *mec*A has major clinical and public health relevance because it encodes PBP2a-mediated methicillin resistance, limits the efficacy of β-lactam therapy, and contributes to MRSA circulation at the animal–human interface. The occurrence of *mec*A-positive *S. aureus* varies across geographic settings, husbandry conditions, antimicrobial use practices, and sampling frames. This variability is evident across published studies: low-frequency MRSA detection with predominantly *mec*A-positive and spa-diverse isolates has been described in Northern Greece [47], whereas raw sheep milk samples collected in Romania showed *mec*A in 13.5% of *S. aureus* isolates together with extensive multi-drug resistance [2]. Higher burdens of *mec*A-positive MRSA and multi-drug resistance have also been documented in other settings [33,48]. In addition, *mec*A-positive staphylococci have been detected in mammary secretions from apparently healthy animals, indicating subclinical reservoirs, and farm-level persistence has been demonstrated even when the apparent prevalence is low [49,50]. These data support the relevance of our findings. In the present study, *mec*A was detected across groups and remained largely stable in the paired analysis, with persistence in most *mecA*-positive pairs and only limited emergence or disappearance after treatment. These results indicate that *mec*A-positive *S. aureus* may remain detectable in the mammary ecosystem across clinically distinct states and after antimicrobial exposure. Clinically, this is important because PBP2a-mediated resistance reduces the expected activity of β-lactam regimens and may contribute to persistence or recurrence. From a public health perspective, these isolates are relevant because they maintain a resistant reservoir at the livestock–human interface and may enter the raw milk chain in systems where milk from treated animals is reintroduced after withdrawal compliance [51,52,53,54]. Overall, even the low-to-moderate detection of *mec*A-positive isolates is epidemiologically relevant because it affects treatment choices, within-flock transmission, and resistance surveillance priorities.

Within this context, the present study provides paired within-animal evidence of the behavior of *mec*A/MRSA around treatment. MRSA-compatible isolates were recovered from both mastitis cases and clinically healthy controls, indicating that *mec*A-positive strains may be maintained at the flock level and are not confined to clinically inflamed udders. Over the short interval between pre-treatment and post-withdrawal sampling, no consistent population-level increase in methicillin resistance was observed: β-lactam resistance profiles remained phenotypically stable, and the paired *mec*A status was predominantly unchanged, with most pairs remaining either negative or persistently positive, whereas emergence and disappearance were limited. When *mec*A emerged, it was generally accompanied by concordant phenotypic methicillin resistance markers, including oxacillin or cefoxitin resistance and/or PBP2a positivity, although limited genotype–phenotype discordance remains biologically plausible because of heteroresistance and variable expression [55,56,57,58]. Overall, these findings indicate that short-term therapy did not measurably increase methicillin resistance at the population level, but existing *mec*A-positive reservoirs persisted. This supports the need for MRSA control strategies in ovine mastitis that combine antimicrobial stewardship with prevention, segregation, milking hygiene, and transmission control, while also limiting opportunities for occupational exposure and dissemination through the raw milk chain [2,33,47,48,50].

In the present study, virulence-associated traits changed more clearly than culture-based outcomes. Enterotoxin detection increased after treatment. Paired analysis supported an increase in SED. SEA and SEB were detected only in post-withdrawal isolates. These findings indicate that the post-treatment *S. aureus* population was enriched in phenotypes with greater toxigenic relevance. Because strain typing was not performed, this pattern cannot be assigned specifically to within-host selection. It is more appropriately interpreted as a shift in the detectable isolated population after treatment. This shift may reflect the persistence of more enterotoxigenic strains, strain turnover, reinfection, or a combination of these processes. The practical implication is clear: milk that re-enters the production chain after withdrawal compliance may still contain *S. aureus* with relevant toxigenic potential, even when overall detection rates remain statistically stable [47,59,60]. This interpretation is consistent with reports from small-ruminant systems showing marked variability in enterotoxigenic profiles across regions and flocks, with SEC often predominant and SEB also present in some settings [43,61,62]. Within this framework, the post-treatment enrichment observed here, especially in group C3, is compatible with the persistence or replacement of strains carrying more relevant virulence traits [56,57,58,63].

A further important finding was the increased frequency of strongly biofilm-adherent isolates after treatment. This may explain why overall culture recovery remained stable while the detectable post-withdrawal population shifted toward persistence-associated phenotypes. Biofilm formation is a recognized determinant of therapeutic failure and chronic *S. aureus* mastitis because biofilm-embedded cells are less susceptible to antimicrobials than planktonic cells [20,64]. Our findings are consistent with ovine and broader ruminant studies linking biofilm capacity to severe disease and variable field expression [41,65,66,67,68]. In the present dataset, the enrichment of strong adherence after treatment may reflect the persistence of highly adherent strains, differential detectability, or strain turnover. Regardless of the mechanism, the post-withdrawal population appears enriched in phenotypes associated with persistence. This supports control strategies that combine antimicrobial stewardship with milking hygiene, the segregation of chronic shedders, reinfection control, and the evaluation of complementary approaches such as phytobiotic-based interventions [69,70].

## 4. Materials and Methods

The milk samples analyzed in this study originated from the same ovine milk collection that has been described comprehensively in our previously published work [71]. Accordingly, the study setting, farm enrollment and animal selection procedures, and clinical case definition are available in [69] and are not reiterated here. However, the objective of this work was not to identify the definitive etiological agent of clinical mastitis, given that mastitis in sheep is often multifactorial, but rather to examine the staphylococcal population profile—with a particular emphasis on *S. aureus* at the species level—in sheep with clinical mastitis receiving a defined antimicrobial treatment protocol, and to evaluate how the recoverable population changed before and after treatment. For clarity, only the key design features required to interpret the staphylococcal results are summarized. Samples represented three clinically defined sampling conditions: group A, clinically healthy udders (controls); group B, clinical mastitis prior to antimicrobial administration (pre-treatment); and group C, resampling of the same mastitis-affected animals at the first eligible milking after completion of the antibiotic agent-specific withdrawal period (post-withdrawal). This structure provided a clinically and temporally resolved framework spanning baseline status, active mastitis, and a post-withdrawal timepoint aligned with milk returning to the processing chain. Clinical mastitis status was determined by veterinary examination based on udder abnormalities and milk alterations (e.g., changes in udder temperature/appearance, pain on palpation, altered milk viscosity/appearance, reduced yield), supported by a California Mastitis Test score of ≥2. Mastitis cases were managed under heightened hygiene, including physical separation from healthy animals. Diagnosis and treatment decisions were made by practicing veterinarians; in field conditions, etiologic identification is often not performed prior to treatment due to logistical and cost constraints, and therapy is commonly selected empirically using clinical presentation and the flock treatment history. It should be noted that the milk produced in most of these farms is given to local dairies, whereas a smaller quantity is processed into artisan cheeses by the families of producers for their own consumption. For each animal, milk samples were aseptically collected separately from each udder half. To minimize the risk of environmental contamination and contamination from the sampler’s hands, strict hygienic precautions were followed throughout the sampling procedure. Sampling was performed by the farm owners under the continuous supervision of the attending veterinarian, in accordance with a standardized protocol that included the use of gloves, cleansing of the udder with lukewarm water, discarding the first three streams of milk, and disinfecting the teat ends with 70% alcohol. Subsequently, 100 mL of milk from each udder half was aseptically collected into sterile Falcon tubes and transported immediately to the laboratory under appropriate conditions for microbiological analysis.

*Clarification of the sampling unit:* Although milk was collected from both udder halves at the same sampling timepoint in all animals, the final microbiological sampling unit was defined at the animal level based on an integrated clinical and laboratory evaluation. Initial mastitis classification was established in the field through veterinary examination, taking into account visible udder abnormalities and changes in milk appearance, and was supported by the California Mastitis Test. This classification was subsequently verified in the laboratory, where the samples were further evaluated and the somatic cell count (SCC) was determined as an additional objective indicator of mastitis status. SCC was measured using a flow cytometric method (CombiFoss 7, Hillerød, Denmark), and only samples with SCC values ≥ 200,000 cells/mL of milk were considered eligible for inclusion in the study. When both udder half-specific samples from the same animal met the mastitis-related inclusion criteria, they were pooled and processed as a single composite sample. In contrast, when mastitis-associated findings were limited to a single udder half, only the sample from the affected half was retained for microbiological analysis, provided that the clinical findings, California Mastitis Test result, and SCC value were all consistent with mastitis. Thus, each animal contributed one microbiologically analyzed sampling unit, corresponding either to a bilateral composite sample or to a unilateral affected-udder sample, according to the confirmed distribution of mastitis-related findings. Overall, 500 animal-level sampling units were included in the microbiological analysis across the study groups.

**Figure 5:**
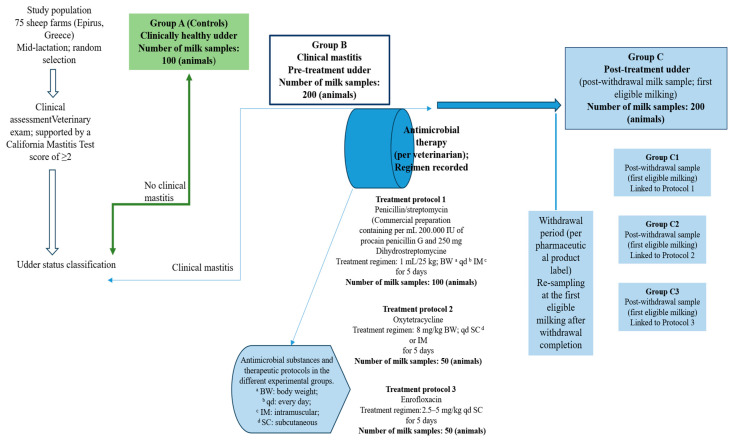
Sampling groups and treatment-linked resampling scheme for *Staphylococcus aureus* isolation. Treatment Protocol 1: penicillin/streptomycin (1 mL/25 kg BW, once daily, IM for 5 days); Treatment Protocol 2: oxytetracycline (8 mg/kg BW, once daily, SC or IM for 5 days); Treatment Protocol 3: enrofloxacin (2.5–5 mg/kg BW, once daily, SC for 5 days). Post-treatment milk was re-collected at the first eligible milking after completion of the product-specific withdrawal period, generating groups C1–C3 corresponding to protocols 1–3, respectively.

A schematic overview (Figure 5) summarizes the grouping and timepoint logic, including the mapping of pre-treatment subgroups (B1–B3) and post-withdrawal subgroups (C1–C3) to the antimicrobial regimens.

### 4.1. Bacterial Isolation—Identification

#### 4.1.1. Culture-Based Methods for Phenotypic Identification of Presumptive *Staphylococcus* spp. Isolates

For microbiological analysis, each milk sample represented a single animal-level sampling unit and had a total volume of 100 or 200 mL, depending on whether one or both udder halves fulfilled the study criteria. Given the relatively large volume, samples were homogenized using a stomacher-type mixing device to achieve an even distribution of bacterial cells. Thereafter, a 10 mL aliquot was aseptically collected with a sterile pipette and added to 90 mL of buffered peptone water (BPW; bioMérieux, Marcy l’Etoile, France), generating the initial 10^−1^ dilution. Serial decimal dilutions were subsequently prepared in sterile Ringer’s solution up to 10^−4^ (1:10,000). To maximize recovery and enhance phenotypic discrimination across the genus *Staphylococcus*, 0.1 mL from each dilution was aseptically plated in parallel onto (i) Baird–Parker agar (Oxoid, London, UK) supplemented with egg yolk tellurite emulsion, a selective medium tailored to the presumptive recovery of coagulase-positive staphylococci (CPS); (ii) mannitol salt agar (Chapman/MSA) for selective/differential screening based on mannitol fermentation; and (iii) 5% sheep blood agar (Columbia blood agar) to evaluate the colony morphology and hemolysis. Plates were incubated aerobically at 35–37 °C for 36–48 h. All distinct colony morphotypes recovered on the three-culture media described above were documented, subcultured to purity, and archived for subsequent analyses. For downstream identification, isolates were screened as presumptive *Staphylococcus* spp. based on the colony morphology, in conjunction with primary microscopy demonstrating Gram-positive cocci in clusters; isolates with phenotypic or microscopic features incompatible with *Staphylococcus* spp. were excluded from further analysis. Presumptive isolates were subsequently subjected to catalase testing and then slide and/or tube coagulase testing to differentiate coagulase-positive staphylococci (CPS) from coagulase-negative staphylococci (CoNS). Presumptive staphylococcal colonies were enumerated, and viable counts were calculated and expressed as colony-forming units per mL (CFU/mL) of sample. The use of three complementary media in parallel, together with the extended incubation and recovery of all distinguishable colony morphotypes, was intended to optimize the detection of phenotypically heterogeneous *Staphylococcus* populations, including colonies with delayed growth or atypical morphologies. Given the recognized capacity of *S. aureus* to form persister-like and small colony-variant subpopulations under stress, this multi-platform recovery strategy was considered appropriate for broad culture-based detection. However, the findings should still be interpreted as referring to the recoverable culturable population under the conditions employed, rather than implying the absolute recovery of all physiologically altered cells [72].

#### 4.1.2. Species Identification

Presumptive *Staphylococcus* isolates were subcultured on 5% sheep Columbia blood agar and incubated for 24 h. Species identification was then performed using matrix-assisted laser desorption/ionization time-of-flight mass spectrometry (MALDI-TOF MS; Bruker Biotyper Microflex, Bruker Daltonik, Bremen, Germany). Spectra were processed with the manufacturer’s software (MBT Compass version 4.1) against the Bruker reference library (BDAL Rev. No 11, 10,833 database entries). Identification confidence was interpreted using Bruker log-score criteria, with scores ≥ 2.0 indicating species-level identification and scores of 1.7–1.99 indicating genus-level identification [73,74].

### 4.2. In Vitro Antimicrobial Susceptibility Tests (AST)

The antimicrobial susceptibility profiles of *S. aureus* isolates were determined by the broth microdilution method in 96-well U-bottom polystyrene microplates using Mueller–Hinton broth (MHB). Minimum inhibitory concentration (MIC) results were interpreted according to the Clinical and Laboratory Standards Institute (CLSI) criteria. The antimicrobial panel was selected to cover the major antibiotic classes and included β-lactams (penicillins: penicillin G, ampicillin, oxacillin; cephalosporins: cefalotin [first generation], ceftiofur [third generation; veterinary-use cephalosporin], and cefquinome [fourth generation; veterinary-use cephalosporin]), glycopeptides (vancomycin, teicoplanin), protein synthesis inhibitors (macrolide: erythromycin; lincosamide: clindamycin; tetracycline: tetracycline; amphenicol: chloramphenicol; aminoglycosides: gentamicin, streptomycin, tobramycin), fluoroquinolones (enrofloxacin, ciprofloxacin), folate pathway antagonists (trimethoprim/sulfamethoxazole), and other agents (fusidic acid). Oxacillin MIC was included as a phenotypic marker supporting the classification of methicillin resistance in *S. aureus*. For inoculum preparation, single well-isolated colonies from overnight culture were suspended in 0.9% saline to match a 0.5 McFarland standard, and the suspension was subsequently diluted to obtain the CLSI-recommended final inoculum per well; then, microplates were incubated at 35 ± 2 °C for 24 h. MIC values were recorded in µg/mL and categorized as susceptible, intermediate, or resistant according to CLSI breakpoints (CLSI, 2020) [75]. ATCC 29213 (methicillin-sensitive control strain) and ATCC 25923 (methicillin-resistant control strain) were used as controls [75,76].

#### 4.2.1. Multiple Antibiotic Resistance (MAR) Indices

To summarize the resistance burden at the isolate level, the multiple antibiotic resistance (MAR) index was calculated for each *S. aureus* isolate as [77]

$$\mathrm{MAR}=\frac{a}{b}$$

where *a* is the number of antimicrobial agents for which the isolate was categorized as resistant, and *b* is the total number of antimicrobial agents tested. Isolates categorized as intermediate were excluded from the MAR calculation (i.e., intermediate results were not counted as resistant in *a* and were excluded from the denominator *b* for that isolate).

To summarize resistance at the epidemiological group level (e.g., by flock, farm, sampling batch, or region), a weighted mean MAR index was computed to account for the distribution of MAR values within each group:

$$\mathrm{Weighted}\;\mathrm{MAR}=\frac{\sum_{i}(n_{i}\times {\mathrm{MAR}}_{i})}{\sum_{i}n_{i}}$$

where $n_{i}$ is the number of isolates in the group sharing MAR value ${\mathrm{MAR}}_{i}$, and $\sum_{i}n_{i}\;$ represents the total number of isolates in that group.

#### 4.2.2. Detection of Methicillin Resistance

Methicillin resistance in the *S. aureus* isolates was evaluated using a tiered phenotypic approach to strengthen classification and to mitigate potential misinterpretation associated with borderline results or the heterogeneous expression of resistance. In addition to the MIC-based β-lactam results reported above, isolates were assessed by cefoxitin disk diffusion, oxacillin salt agar screening (OSAS), and PBP2a latex slide agglutination, thereby integrating a robust surrogate marker (cefoxitin), a selective growth screen (OSAS), and a mechanism-oriented confirmation of PBP2a expression.

Cefoxitin disk diffusion (DD) was performed according to the CLSI reference method using 30 µg cefoxitin disks (Oxoid, Basingstoke, UK) on Mueller–Hinton agar, with inhibition zones read after 18–24 h at 35 °C and interpreted using CLSI criteria [75,78].

For the oxacillin salt agar screen (OSAS), commercially prepared Mueller–Hinton agar plates (MHA) supplemented with 4% NaCl and oxacillin at 6, 1, or 0.75 µg/mL were used. Approximately 10^4^ CFUs were spot-inoculated onto each plate and incubated for 24 h at 35 °C. Any visible growth on oxacillin-containing medium was considered a positive screen.

PBP2a was detected using a rapid latex slide agglutination assay (Slidex MRSA Detection Test; bioMérieux, Paris, France), following the manufacturer’s instructions. Briefly, bacterial biomass from a fresh culture was extracted using the kit reagents with heat treatment, the extract was clarified by centrifugation (1500× *g*, 5 min), and the supernatant was tested with anti-PBP2a antibody-sensitized latex particles alongside the provided negative control latex. Agglutination reactions were read at approximately 10 min (and up to 15 min when required by the kit guidance).

#### 4.2.3. Inducible Resistance to Clindamycin

Inducible clindamycin resistance was assessed to identify *erm*-mediated MLSB phenotypes that may not be apparent from routine clindamycin results but can compromise clindamycin efficacy. Testing was performed by the CLSI D-test (disk approximation method) on Mueller–Hinton agar using a lawn inoculum standardized to 0.5 McFarland. Erythromycin and clindamycin disks were placed 15 mm apart (edge-to-edge) and plates were incubated at 35–37 °C overnight. D-shaped blunting of the clindamycin inhibition zone adjacent to the erythromycin disk was interpreted as inducible clindamycin resistance, whereas a circular clindamycin zone in the presence of erythromycin resistance was interpreted as a D-test-negative result, in accordance with CLSI guidance [75,79].

### 4.3. Phenotypic Assessment of Virulence-Associated Traits

#### 4.3.1. Staphylococcal Enterotoxin Reverse Passive Latex Agglutination Kit (SET-RPLA) Phenotypic Ability to Produce (RPLA Method)

Staphylococcal enterotoxin production was assessed using the Reverse Passive Latex Agglutination (SET-RPLA) kit (Oxoid, Hampshire, UK), which employs monoclonal anti-enterotoxin antibodies and detects the classical staphylococcal enterotoxins A–D (SEA–SED) at concentrations down to 0.5 ng/mL [80,81].

#### 4.3.2. Biofilm Formation Assay—Microtiter Plate Biofilm (Adherence) Assay and Interpretation

Biofilm formation was quantified using a static 96-well flat-bottom polystyrene microtiter plate assay with crystal violet staining as an endpoint measure of the total attached biomass. Overnight *S. aureus* cultures were grown in tryptic soy broth (TSB) supplemented with 1% glucose and adjusted to a 0.5 McFarland standard. Each well was inoculated with 200 µL of the standardized suspension; wells containing broth alone served as negative controls. Plates were covered and incubated aerobically at 37 °C for 24 h under static conditions to allow surface attachment and biofilm development. After incubation, the planktonic phase was discarded, and wells were gently washed three times with 250 µL sterile physiological saline to remove non-adherent cells, with brief shaking after each wash to improve the removal of loosely attached bacteria. The remaining attached biomass was fixed with 200 µL of 99% methanol per well for 20 min. Plates were then emptied and air-dried at room temperature. Adherent cells were stained with 200 µL of 2% (*w*/*v*) crystal violet for 5 min. Excess stain was removed by thorough rinsing with water, and plates were air-dried. Bound dye was solubilized with 160 µL of 33% (*v*/*v*) glacial acetic acid per well, and absorbance was measured at 570 nm using a microplate reader. Where baseline readings were collected prior to dye solubilization, only the OD_570_ measured after acetic acid resolubilization was used for biofilm quantification [82,83,84].

To standardize the interpretation of biofilm biomass, a plate-specific cut-off optical density (OD*_c_*) was calculated from the negative control wells (broth only) as

$${\mathrm{OD}}_{c}={\overset{‾}{\mathrm{OD}}}_{\mathrm{negative}\;\mathrm{control}}+3\times {\mathrm{SD}}_{\mathrm{negative}\;\mathrm{control}}$$

where SD: standard deviation.

Using OD*_c_*, each isolate was categorized based on its OD_570_ as follows (Christensen- type classification [85]): non-adherent: $\mathrm{OD}\leq {\mathrm{OD}}_{c}$; weakly adherent: ${\mathrm{OD}}_{c}<\mathrm{OD}\leq 2\times {\mathrm{OD}}_{c}$; moderately adherent: $2\times {\mathrm{OD}}_{c}<\mathrm{OD}\leq 4\times {\mathrm{OD}}_{c}$; strongly adherent: $\mathrm{OD}>4\times {\mathrm{OD}}_{c}$.

This approach accounts for plate-to-plate background variation and enables a consistent comparison of adherence phenotypes across assay runs. All tests were performed in triplicate, and the results were reported as the mean OD_570_ (and corresponding adherence category) for each isolate.

### 4.4. Detection of mecA Gene in S. aureus Isolates

To complement phenotypic testing and strengthen the interpretation of the methicillin resistance profiles relevant to mastitis epidemiology and milk safety, *S. aureus* isolates were screened by PCR for the *mec*A gene, a major determinant of PBP2a-mediated methicillin resistance. Genomic DNA was extracted using the DNeasy^®^ UltraClean^®^ Microbial Kit (Qiagen Inc., Toronto, ON, Canada), according to the manufacturer’s instructions, and stored at −20 °C until analysis. Reactions (25 µL) contained 5 µL template DNA, 1 µL each of the forward and reverse primers, 12 µL of 2× PCR master mix (Qiagen), and 6 µL of RNase-free water. Amplification was carried out in a CFX96™ thermal cycler (Bio-Rad, Hercules, CA, USA). PCR products were resolved on 2% agarose gels, stained with ethidium bromide, and visualized under UV illumination. A 100 bp DNA ladder was used as a size marker, and a no-template control was included in every run to monitor for contamination. The detection of *mec*A was performed using the following primer pair (5′ → 3′): *mec*A-F: TCCAGATTACAACTTCACCAGG and *mec*A-R: CCACTTCATATCTTGTAACG (amplicon length 162 bp) [86,87]. The thermal cycling conditions were as follows: initial denaturation at 95 °C for 5 min, followed by 35 cycles of 95 °C for 30 s, 55.7 °C for 30 s, and 72 °C for 45 s, with a final extension at 72 °C for 5 min.

### 4.5. Statistical Analysis

Data were analyzed in SPSS v25. Categorical outcomes (e.g., *S. aureus* detection, resistance/virulence positivity, *mec*A status) were compared using χ^2^ or Fisher’s exact tests, as appropriate. For within-animal pre–post comparisons (paired isolates), McNemar’s test was applied. Continuous/ordinal variables (e.g., CFU categories, MAR indices, biofilm adherence scores) were evaluated with non-parametric paired or independent tests when distributional assumptions were not met. Differences in class resistance prevalence were assessed using Fisher’ s exact test with Holm correction within each class. In parallel, a continuous within-class burden measure (class MAR = number resistant within class/number tested within class) was compared across groups using the Kruskal–Wallis (KW) test. Changes in phenotypic enterotoxin detection were assessed by McNemar’s test. Paired (within-animal) comparisons were performed between the pre-treatment mastitis groups (B1–B3) and the corresponding post-treatment groups (C1–C3) by sign tests and Willcoxon sign tests. All tests were two-sided, and statistical significance was set at *p* < 0.05.

## 5. Limitations of This Study

This study was conducted in sheep flocks from Epirus, Greece. This locality exhibits a specific productive system, which is a significant parameter that affects the bacterial diversity of the territorial ecosystem as well as the microecosystems in each farm, with distinct characteristics in terms of population dynamics and the resistome burden.

Treatment allocation was performed under field conditions by the attending veterinarians and was not randomized. For this reason, comparisons between therapeutic protocols should be interpreted as observational rather than causal. Differences between protocols may reflect both treatment effects and underlying clinical or management differences between the treated animals.

Post-treatment sampling was performed only once, at the first eligible milking after the withdrawal period. This timepoint is directly relevant to food chain assessment. However, it does not allow an evaluation of medium-term persistence, recurrence, intermittent shedding, or delayed post-treatment changes. Repeated sampling after treatment would provide a more complete picture.

The study relied on culture-based recovery and threshold-based categorization of the bacterial burden. A negative culture at a single timepoint does not exclude low-level carriage or intermittent shedding. Likewise, transitions between negative and positive status may reflect biological fluctuations around the culture detection threshold. This limitation should be considered when interpreting the paired pre- and post-treatment comparisons.

The number of *mec*A-positive isolates was limited. The number of paired baseline-positive animals was also small, especially in therapeutic protocols 2 and 3. This reduced the power to evaluate treatment-related changes in methicillin resistance dynamics and other paired outcomes.

Finally, apart from the detection of the resistance determinant *mec*A, the evaluation of antimicrobial susceptibility was based mainly on phenotypic characterization. Strain-level molecular typing was not performed. As a result, post-treatment isolates could not be distinguished with certainty as the persistence of the same strain, the emergence of previously undetected subpopulations, or strain replacement/reinfection. Molecular analysis of toxin genes, biofilm-associated determinants, and clonal relatedness would strengthen the interpretation of the post-treatment shifts observed in resistance, enterotoxigenicity, and biofilm-associated phenotypes.

## 6. Conclusions

In this field study of ewe mammary secretions and raw milk from 75 sheep farms (depending on the study endpoint), *S. aureus* behaved as an endemic component of the flock microbiological ecosystem. It was recovered at broadly comparable frequencies from clinically healthy controls, clinical mastitis cases before treatment, and the same animals at the first milking after withdrawal compliance, indicating that *S. aureus* is not confined to overt udder inflammation but is sustained by a wider reservoir of subclinical carriage and intermittent shedding. Within this ecological setting, antimicrobial intervention did not translate into a measurable short-term shift in culture-based recovery at the post-withdrawal timepoint, yet persistent culture positivity occurred under all protocols, underscoring that a bacteriological cure is not assured by the time at which milk becomes legally eligible to re-enter the market and that control cannot be inferred from “detected/not detected” outcomes alone.

Methicillin resistance determinants were present across groups, supporting the notion that *mec*A-positive/MRSA-compatible subpopulations may be maintained at the flock level and can persist through therapy. Paired comparisons showed that the *mec*A status was largely stable, with only uncommon emergence or loss, consistent with resistance determinants that are not rapidly cleared over short follow-up intervals and with the likelihood that post-treatment isolates may reflect persistence and/or strain turnover. Given the therapeutic constraints imposed by PBP2a-mediated β-lactam resistance, these *mec*A-positive profiles remain clinically relevant for mastitis management and important for antimicrobial resistance surveillance at the livestock–human interface.

Overall, the findings support a cautious but actionable inference: antimicrobial therapy alone is unlikely to ensure eradication and may leave residual populations enriched for persistence and hazard traits. Effective risk reduction therefore requires integrated flock-level control—milking hygiene, segregation/management of chronic shedders, reinfection prevention, and surveillance that includes resistance determinants and virulence/persistence phenotypes—alongside prudent antimicrobial selection.

## Figures and Tables

**Table 1:** Classification of experimental groups, clinical status, sampling timepoints, and treatment allocation in the study population.

Group	Experimental Group Classification ^a^	Clinical Status	Sampling Timepoint	Treatment		n ^b^	Notes
Group A	A	Clinically healthy udders	Baseline/control sampling	None		100	Control group
Group B	B	Clinical mastitis	Before treatment	Not yet treated		200	Pre-treatment mastitis group
	B1	Clinical mastitis	Before treatment	Protocol 1 ^c^	Penicillin G/dihydrostreptomycin	100	Subgroup of Group B
	B2	Clinical mastitis	Before treatment	Protocol 2 ^d^	Oxytetracycline	50	Subgroup of Group B
	B3	Clinical mastitis	Before treatment	Protocol 3 ^e^	Enrofloxacin	50	Subgroup of Group B
Group C	C1	Post-treatment, corresponding to B1 animals	First eligible milking after withdrawal period	Protocol 1	Penicillin G/dihydrostreptomycin	100	Same animals as B1
	C2	Post-treatment, corresponding to B2 animals	First eligible milking after withdrawal period	Protocol 2	Oxytetracycline	50	Same animals as B2
	C3	Post-treatment, corresponding to B3 animals	First eligible milking after withdrawal period	Protocol 3	Enrofloxacin	50	Same animals as B3

^a^: Experimental group classification was based on udder clinical status and sampling timepoint. ^b^: n = number of animal-level sampling units. ^c^: Therapeutic protocol 1: penicillin G/dihydrostreptomycin. ^d^: Therapeutic protocol 2: oxytetracycline. ^e^: Therapeutic protocol 3: enrofloxacin. “Same animals” denotes paired sampling of the same mastitis-affected animals before treatment (group B) and after completion of the corresponding withdrawal period (group C).

**Table 2:** Distribution of *S. aureus* viable counts (log_10_ CFU/mL) in milk samples by study group, timepoint, and treatment protocol (according to the antibiotic administered).

Experimental Group *	n	No Growth (0 **), n (%)	Detected <1.00, n (%)	Quantified ≥1.00, n (%)	Detected (Any), n (%)	Quantified Load (≥1.00 Only), Median (IQR ***); n
A (controls; clinically healthy udders)	100	77 (77.0)	3 (3.0)	20 (20.0)	23 (23.0)	2.06 (1.00–2.22); 20
B1	100	70 (70.0)	8 (8.0)	22 (22.0)	30 (30.0)	2.55 (2.31–3.33); 22
C1	100	78 (78.0)	9 (9.0)	13 (13.0)	22 (22.0)	2.10 (1.58–2.54); 13
B2	50	39 (78.0)	5 (10.0)	6 (12.0)	11 (22.0)	2.37 (2.09–2.69); 6
C2	50	39 (78.0)	5 (10.0)	6 (12.0)	11 (22.0)	2.39 (2.06–2.74); 6
B3	50	38 (76.0)	2 (4.0)	10 (20.0)	12 (24.0)	1.94 (1.00–2.06); 10
C3	50	40 (80.0)	3 (6.0)	7 (14.0)	10 (20.0)	2.10 (1.98–2.27); 7

*: The definition and interpretation of the sampled groups is provided in Section 4 (Materials & Methods) and in the preceding text, which introduces the animal groups and summarizes the characteristics of each sampled group. **: Interpretation of culture quantification: 0 = no growth; <1.00 log_10_ CFU/mL = detected but below the quantification threshold (<10 CFU/mL). Detected (any) includes both non-quantifiable (<1.00) and quantifiable (≥1.00) results. Quantified load summaries are reported only among quantified positives (≥1.00). ***: IQR, interquartile range; quantified load values are presented as median (IQR), where IQR denotes the interquartile range.

**Table 3:** The distribution of antimicrobial resistance phenotypes of the isolated *S. aureus* strains in the different groups by mode of action of the tested antibacterial substance.

Antimicrobial Agent	Experimental Group																				
	A ^a^ (n ^b^ = 22)			B1 (n = 30)			C1 (n = 22)			B2 (n = 11)			C2 (n = 11)			B3 (n = 12)			C3 (n = 7)		
	S ^c^	I	R	S	I	R	S	I	R	S	I	R	S	I	R	S	I	R	S	I	R
Mechanism of action: Inhibition of cell wall synthesis (β-lactams)																					
BP ^d^	14 (63.6)	0 (0.0)	8 (36.4)	20 (66.7)	0 (0.0)	10 (33.3)	2 (9.1)	0 (0.0)	20 (90.9)	0 (0.0)	0 (0.0)	11 (100.0)	1 (9.1)	0 (0.0)	10 (90.9)	0 (0.0)	0 (0.0)	12 (100.0)	0 (0.0)	0 (0.0)	7 (100.0)
AM	14 (63.6)	0 (0.0)	8 (36.4)	15 (50.0)	0 (0.0)	15 (50.0)	5 (22.7)	0 (0.0)	17 (77.3)	0 (0.0)	0 (0.0)	11 (100.0)	0 (0.0)	0 (0.0)	11 (100.0)	1 (8.3)	0 (0.0)	11 (91.7)	0 (0.0)	0 (0.0)	7 (100.0)
OX	17 (77.3)	0 (0.0)	5 (22.7)	24 (80.0)	0 (0.0)	6 (20.0)	13 (59.1)	0 (0.0)	9 (40.9)	11 (100.0)	0 (0.0)	0 (0.0)	11 (100.0)	0 (0.0)	0 (0.0)	9 (75.0)	0 (0.0)	3 (25.0)	6 (85.7)	0 (0.0)	1 (14.3)
CE	5 (22.7)	6 (27.3)	11 (50.0)	0 (0.0)	3 (10.0)	27 (90.0)	1 (4.5)	3 (13.6)	18 (81.8)	4 (36.4)	0 (0.0)	7 (63.6)	6 (54.5)	0 (0.0)	5 (45.5)	9 (75.0)	0 (0.0)	3 (25.0)	4 (57.1)	0 (0.0)	3 (42.9)
CEF	14 (63.6)	3 (13.6)	5 (22.7)	15 (50.0)	5 (16.7)	10 (33.3)	7 (31.8)	1 (4.5)	14 (63.6)	6 (54.5)	0 (0.0)	5 (45.5)	8 (72.7)	3 (27.3)	0 (0.0)	8 (66.7)	0 (0.0)	4 (33.3)	4 (57.1)	1 (14.3)	2 (28.6)
CFQ	21 (95.5)	0 (0.0)	1 (4.5)	26 (86.7)	0 (0.0)	4 (13.3)	17 (77.3)	0 (0.0)	5 (22.7)	9 (81.8)	0 (0.0)	2 (18.2)	10 (90.9)	0 (0.0)	1 (9.1)	9 (75.0)	0 (0.0)	3 (25.0)	6 (85.7)	0 (0.0)	1 (14.3)
Mechanism of action: Glycopeptides (cell wall synthesis)																					
VN	19 (86.4)	3 (13.6)	0 (0.0)	27 (90.0)	1 (3.3)	2 (6.7)	19 (86.4)	1 (4.5)	2 (9.1)	10 (90.9)	0 (0.0)	1 (9.1)	9 (81.8)	0 (0.0)	2 (18.2)	11 (91.7)	1 (8.3)	0 (0.0)	7 (100.0)	0 (0.0)	0 (0.0)
TP	22 (100.0)	0 (0.0)	0 (0.0)	30 (100.0)	0 (0.0)	0 (0.0)	22 (100.0)	0 (0.0)	0 (0.0)	11 (100.0)	0 (0.0)	0 (0.0)	10 (90.9)	1 (9.1)	0 (0.0)	12 (100.0)	0 (0.0)	0 (0.0)	7 (100.0)	0 (0.0)	0 (0.0)
Mechanism of action: Protein synthesis inhibitors—50S ribosomal subunit																					
ER	15 (68.2)	1 (4.5)	6 (27.3)	28 (93.3)	1 (3.3)	1 (3.3)	22 (100.0)	0 (0.0)	0 (0.0)	8 (72.7)	0 (0.0)	3 (27.3)	8 (72.7)	0 (0.0)	3 (27.3)	12 (100.0)	0 (0.0)	0 (0.0)	7 (100.0)	0 (0.0)	0 (0.0)
CL	17 (77.3)	3 (13.6)	2 (9.1)	18 (60.0)	11 (36.7)	1 (3.3)	22 (100.0)	0 (0.0)	0 (0.0)	11 (100.0)	0 (0.0)	0 (0.0)	10 (90.9)	1 (9.1)	0 (0.0)	12 (100.0)	0 (0.0)	0 (0.0)	7 (100.0)	0 (0.0)	0 (0.0)
CHL	20 (90.9)	1 (4.5)	1 (4.5)	26 (86.7)	0 (0.0)	4 (13.3)	21 (95.5)	0 (0.0)	1 (4.5)	11 (100.0)	0 (0.0)	0 (0.0)	8 (72.7)	0 (0.0)	3 (27.3)	12 (100.0)	0 (0.0)	0 (0.0)	6 (85.7)	1 (14.3)	0 (0.0)
Mechanism of action: Protein synthesis inhibitors—30S ribosomal subunit																					
TE	13 (59.1)	0 (0.0)	9 (40.9)	25 (83.3)	0 (0.0)	5 (16.7)	22 (100.0)	0 (0.0)	0 (0.0)	7 (63.6)	0 (0.0)	4 (36.4)	5 (45.5)	0 (0.0)	6 (54.5)	6 (50.0)	0 (0.0)	6 (50.0)	5 (71.4)	0 (0.0)	2 (28.6)
GE	20 (90.9)	1 (4.5)	1 (4.5)	30 (100.0)	0 (0.0)	0 (0.0)	22 (100.0)	0 (0.0)	0 (0.0)	11 (100.0)	0 (0.0)	0 (0.0)	11 (100.0)	0 (0.0)	0 (0.0)	12 (100.0)	0 (0.0)	0 (0.0)	7 (100.0)	0 (0.0)	0 (0.0)
ST	20 (90.9)	0 (0.0)	2 (9.1)	27 (90.0)	1 (3.3)	2 (6.7)	19 (86.4)	0 (0.0)	3 (13.6)	11 (100.0)	0 (0.0)	0 (0.0)	11 (100.0)	0 (0.0)	0 (0.0)	10 (83.3)	0 (0.0)	2 (16.7)	7 (100.0)	0 (0.0)	0 (0.0)
TOB	21 (95.5)	1 (4.5)	0 (0.0)	30 (100.0)	0 (0.0)	0 (0.0)	22 (100.0)	0 (0.0)	0 (0.0)	11 (100.0)	0 (0.0)	0 (0.0)	11 (100.0)	0 (0.0)	0 (0.0)	12 (100.0)	0 (0.0)	0 (0.0)	7 (100.0)	0 (0.0)	0 (0.0)
QD	22 (100.0)	0 (0.0)	0 (0.0)	28 (93.3)	0 (0.0)	2 (6.7)	20 (90.9)	1 (4.5)	1 (4.5)	7 (63.6)	2 (18.2)	2 (18.2)	9 (81.8)	0 (0.0)	2 (18.2)	9 (75.0)	2 (16.7)	1 (8.3)	5 (71.4)	0 (0.0)	2 (28.6)
Mechanism of action: Inhibition of DNA gyrase (fluoroquinolones)																					
EN	16 (72.7)	4 (18.2)	2 (9.1)	30 (100.0)	0 (0.0)	0 (0.0)	22 (100.0)	0 (0.0)	0 (0.0)	11 (100.0)	0 (0.0)	0 (0.0)	11 (100.0)	0 (0.0)	0 (0.0)	11 (91.7)	0 (0.0)	1 (8.3)	3 (42.9)	0 (0.0)	4 (57.1)
CIP	18 (81.8)	1 (4.5)	3 (13.6)	29 (96.7)	1 (3.3)	0 (0.0)	20 (90.9)	2 (9.1)	0 (0.0)	11 (100.0)	0 (0.0)	0 (0.0)	11 (100.0)	0 (0.0)	0 (0.0)	11 (91.7)	1 (8.3)	0 (0.0)	4 (57.1)	0 (0.0)	3 (42.9)
Mechanism of action: Inhibition of tetrahydrofolic acid synthesis																					
Tr/Sulf	19 (86.4)	0 (0.0)	3 (13.6)	30 (100.0)	0 (0.0)	0 (0.0)	22 (100.0)	0 (0.0)	0 (0.0)	9 (81.8)	0 (0.0)	2 (18.2)	11 (100.0)	0 (0.0)	0 (0.0)	12 (100.0)	0 (0.0)	0 (0.0)	5 (71.4)	0 (0.0)	2 (28.6)
Other agents																					
FcA	20 (90.9)	0 (0.0)	2 (9.1)	29 (96.7)	0 (0.0)	1 (3.3)	21 (95.5)	0 (0.0)	1 (4.5)	11 (100.0)	0 (0.0)	0 (0.0)	10 (90.9)	0 (0.0)	1 (9.1)	12 (100.0)	0 (0.0)	0 (0.0)	5 (71.4)	0 (0.0)	2 (28.6)

^a^: Experimental groups; ^b^: number of *S. aureus* strains isolated in the experimental group; ^c^: S, I, R for susceptible, intermediate, and resistant strains, respectively, according to the CLSI guidance values for MIC; ^d^: penicillin G (BP), ampicillin (AM), oxacillin (OX), cefalotin (CE), ceftiofur (CEF), cefquinome (CFQ), vancomycin (VN), teicoplanin (TP), erythromycin (ER), clindamycin (CL), tetracycline (TE), chloramphenicol (CHL), gentamicin (GE), streptomycin (ST), tobramycin (TOB), quinupristin-dalfopristin (QD), enrofloxacin (EN), ciprofloxacin (CIP), trimethoprim/sulfamethoxazole (Tr/Sulf), fusidic acid (FcA).

**Table 4:** Multiple antibiotic resistance (MAR) and weighted MAR indices of *S. aureus* isolates across control (A), pooled pre-treatment mastitis (B), and protocol-specific pre-/post-treatment groups (B1–C3).

Experimental Group	n ^a^	Antibiotics Tested	MAR ^b^ Mean ± SD	MAR Median [“IQR ^c^”]	Weighted MAR ^d^ Mean ± SD	Weighted MAR Median [“IQR”]
A	22	20	0.157 ± 0.111	0.150 [0.100–0.150]	0.120 ± 0.098	0.097 [0.053–0.156]
B (pooled pre-treatment)	51	20	0.175 ± 0.099	0.150 [0.100–0.250]	0.111 ± 0.080	0.083 [0.056–0.135]
B1	30	20	0.150 ± 0.103	0.150 [0.062–0.188]	0.091 ± 0.074	0.079 [0.034–0.129]
C1	22	20	0.207 ± 0.085	0.200 [0.150–0.250]	0.124 ± 0.058	0.111 [0.089–0.139]
B2	10	20	0.225 ± 0.079	0.250 [0.150–0.300]	0.167 ± 0.092	0.173 [0.080–0.221]
C2	11	20	0.200 ± 0.045	0.200 [0.200–0.225]	0.132 ± 0.048	0.107 [0.103–0.177]
B3	11	20	0.200 ± 0.089	0.200 [0.150–0.225]	0.114 ± 0.062	0.107 [0.079–0.121]
C3	7	20	0.271 ± 0.125	0.200 [0.200–0.350]	0.277 ± 0.164	0.222 [0.167–0.373]

^a^: Number of *S. aureus* strains isolated in the experimental group; ^b^: MAR = (number of antibiotics resistant)/20; ^c^: IQR: interquartile range, defined as the range between the 25th and 75th percentiles; ^d^: weighted MAR was calculated as the mean of within-class resistance proportions, giving equal weight to each antimicrobial class; details are provided in Section 4.2.

**Table 5:** Class-level resistance prevalence and resistance burden testing (Class MAR) among *S. aureus* isolates from control animals and mastitis groups before and after therapy.

Class	Comparison Between Experimental Groups	R/N ^a^ (%) Group 1 ^b^	R/N (%) Group 2	*p* (Holm)	KW *p* (Class MAR)
β-lactams	Group A vs. Group B (combined pre-treatment mastitis group)	17/22 (77.3%)	50/51 (98.0%)	0.03331	0.0002768
Fluoroquinolones	Group A vs. Group B (combined pre-treatment mastitis group)	5/22 (22.7%)	1/51 (2.0%)	0.02498	1.345 × 10^−8^
Fluoroquinolones	Group B (combined pre-treatment mastitis group) vs. Group C3	1/51 (2.0%)	5/7 (71.4%)	0.0001065	1.345 × 10^−8^
Other agents	Group B (combined pre-treatment mastitis group) vs. Group C3	5/51 (9.8%)	4/7 (57.1%)	0.03291	0.007963

^a^: R/N (%) denotes the number of isolates classified as resistant within the indicated antimicrobial class divided by the total number of isolates in the comparison, with the corresponding percentage in parentheses; ^b^: Group 1 and Group 2 correspond to the first and second groups listed in the “Comparison between experimental groups” column. Differences in class resistance prevalence were assessed using Fisher’s exact test, and *p* (Holm) denotes the *p*-value after Holm correction for multiple comparisons within each class. Class MAR (class-specific multiple antibiotic resistance index) was calculated per isolate as (number of resistant antibiotics within the class)/(number tested within the class); KW *p* (Class MAR) is the Kruskal–Wallis *p*-value for overall differences in Class MAR across A, B (pooled, combined pre-treatment mastitis group), C1, C2, and C3. Two-sided *p*-values < 0.05 were considered statistically significant.

**Table 6:** Distribution of methicillin resistance markers and composite MRSA classification among *S. aureus* isolates across experimental groups.

Phenotypic Screening Index/Genotyping	Experimental Group/Total Number of *S. aureus* Isolates						
	A (n ^a^ = 22)	B1 (n = 30)	C1 (n = 22)	B2 (n = 11)	C2 (n = 11)	B3 (n = 12)	C3 (n = 7)
**OX^®^** (oxacillin MIC resistant) *	5 ^b^ (22.7 ^b^)	6 (20.0)	9 (40.9)	0 (0.0)	0 (0.0)	3 (25.0)	1 (14.3)
**Cefoxitin_pos** (DD zone positive) **	4 (18.2)	5 (16.7)	6 (27.3)	3 (27.3)	3 (27.3)	4 (33.3)	2 (28.6)
**OSAS pos** (any OSAS asay positive) ***	3 (13.6) ^b^	6 (20.0)	8 (36.4)	2 (18.2)	1 (9.1)	4 (33.3)	2 (28.6)
PBP2a_pos ****	4 (18.2)	11 (36.7)	16 (72.7)	3 (27.3)	1 (9.1)	4 (33.3)	2 (28.6)
mecA_pos *****	3 (13.6)	10 (33.3)	12 (54.5)	5 (45.5)	2 (18.2)	5 (41.7)	3 (42.9)
**MRSA_screen** (composite)	7 (31.8)	15 (50.0)	19 (86.4)	3 (27.3)	3 (27.3)	5 (41.7)	2 (28.6)
**MRSA_confirmed** (composite)	3/22 (13.6)	10/30 (33.3)	12/22 (54.5)	3/11 (27.3)	2/11 (18.2)	5/12 (41.7)	2/7 (28.6)

^a^: Number of *S. aureus* strains isolated in the experimental group; ^b^: number (percentage) of assay-positive *S. aureus* isolates; *: oxacillin MIC-resistant isolates; **: cefoxitin disk diffusion positivity; ***: positivity in any oxacillin salt agar screening assay; ****: detection of penicillin-binding protein 2a (PBP2a); *****: *mec*A gene detection. MRSA_screen (composite) = isolate positive for at least one methicillin resistance screening marker included in the study; MRSA_confirmed (composite) = isolate classified as methicillin-resistant *S. aureus* on the basis of confirmatory evidence from the composite phenotypic/genotypic assessment applied in this study.

**Table 7:** Phenotypic detection of classical staphylococcal enterotoxins (SEA–SED) and overall enterotoxigenicity of *S. aureus* isolates by experimental group.

Experimental Group	n ^a^	Toxin Serotype				
		SEA ^c^	SEB	SEC	SED	Enterotoxigenic (≥1 Toxin) ^d^
A	22	3 ^b^/22 (13.6%)	2/22 (9.1%)	4/22 (18.2%)	2/22 (9.1%)	8/22 (36.4%)
B1	30	0/30 (0.0%)	0/30 (0.0%)	5/30 (16.7%)	5/30 (16.7%)	9/30 (30.0%)
B2	11	0/11 (0.0%)	0/11 (0.0%)	2/11 (18.2%)	2/11 (18.2%)	4/11 (36.4%)
B3	12	0/12 (0.0%)	0/12 (0.0%)	2/12 (16.7%)	1/12 (8.3%)	3/12 (25.0%)
C1	22	3/22 (13.6%)	0/22 (0.0%)	5/22 (22.7%)	7/22 (31.8%)	10/22 (45.5%)
C2	11	0/11 (0.0%)	2/11 (18.2%)	2/11 (18.2%)	3/11 (27.3%)	5/11 (45.5%)
C3	7	1/7 (14.3%)	1/7 (14.3%)	3/7 (42.9%)	4/7 (57.1%)	6/7 (85.7%)

^a^: Number of *S. aureus* strains isolated in the experimental group. ^b^: Data are presented as the number of positive isolates, with the corresponding percentage in parentheses for each enterotoxin serotype. ^c^: SEs (SEA–SED), SEA: Staphylococcal Enterotoxin A, SEB: Staphylococcal Enterotoxin B, SEC: Staphylococcal Enterotoxin C; SED: Staphylococcal Enterotoxin D. ^d^: Positive if the isolate is positive for at least one of the four studied enterotoxins (SEA, SEB, SEC, or SED) by SET-RPLA.

## Data Availability

Data are contained within the article.

## Supplementary Materials

The following supporting information can be downloaded at https://www.mdpi.com/article/10.3390/antibiotics15040388/s1, Table S1: Paired pre- to post-treatment changes in phenotypic detection of classical staphylococcal enterotoxins (SEA–SED) in Staphylococcus aureus isolates, overall and by therapeutic protocol (exact McNemar test).
