# Supplementary material for: Ecological Dynamics of Staphylococcus aureus in Raw Ewe Milk Following Different Mastitis Treatment Protocols
Source: Antibiotics (Basel). 2026 Apr 10;15(4):388. doi: 10.3390/antibiotics15040388 (PMC13113595; doi:10.3390/antibiotics15040388)
Supplement: Supplementary file 1 [file antibiotics-15-00388-s001.zip › S1.pdf]

Table S1: Paired pre- to post-treatment changes in phenotypic detection of classical staphylococcal enterotoxins (SEA–SED) in *Staphylococcus aureus* isolates, overall and by therapeutic protocol (exact McNemar test)

Comparison	Index	Paired n[a]	Transition pattern				McNemar[f] *p*
			Persistent positivity[b]	Loss of positivity after treatment[c]	Gain of positivity after treatment[d]	Persistent negativity[e]	
Overall: B→C (all protocols)	SEA[g]	19	0	0	1	18	1.000
Overall: B→C (all protocols)	SEB	19	0	0	0	19	NA
Overall: B→C (all protocols)	SEC	19	7	0	2	10	0.500
Overall: B→C (all protocols)	SED	19	1	0	10	8	0.002
Overall: B→C (all protocols)	ANY[h]	19	7	0	5	7	0.062
B1→C1 (Protocol 1)	SEA	11	0	0	1	10	1.000
B1→C1 (Protocol 1)	SEB	11	0	0	0	11	NA[i]
B1→C1 (Protocol 1)	SEC	11	3	0	1	7	1.000
B1→C1 (Protocol 1)	SED	11	1	0	4	6	0.125
B1→C1 (Protocol 1)	ANY	11	3	0	3	5	0.250
B2→C2 (Protocol 2)	SEA	3	0	0	0	3	NA
B2→C2 (Protocol 2)	SEB	3	0	0	0	3	NA
B2→C2 (Protocol 2)	SEC	3	2	0	0	1	NA
B2→C2 (Protocol 2)	SED	3	0	0	2	1	0.500
B2→C2 (Protocol 2)	ANY	3	2	0	0	1	NA
B3→C3 (Protocol 3)	SEA	5	0	0	0	5	NA
B3→C3 (Protocol 3)	SEB	5	0	0	0	5	NA
B3→C3 (Protocol 3)	SEC	5	2	0	1	2	1.000
B3→C3 (Protocol 3)	SED	5	0	0	4	1	0.125
B3→C3 (Protocol 3)	ANY	5	2	0	2	1	0.500

Footnotes:
[a]: paired n = number of paired isolates available for within-animal comparison between the pre-treatment and post-treatment sampling points
[b]: Persistent positivity = positive before treatment and positive after treatment
[c]: Loss of positivity = positive before treatment and negative after treatment
[d]: Gain of positivity = negative before treatment and positive after treatment
[e]: Persistent negativity = negative before treatment and negative after treatment
[f]: McNemar p = exact McNemar test p-value calculated from the discordant paired outcomes only, that is, loss of positivity versus gain of positivity
[g]: SEA, staphylococcal enterotoxin A; SEB, staphylococcal enterotoxin B; SEC, staphylococcal enterotoxin C; SED, staphylococcal enterotoxin D
[h]: ANY = positivity for at least one of the four classical enterotoxins tested (SEA, SEB, SEC, or SED)
[i]: NA = not applicable; the test was not performed because no discordant paired outcomes were present.
